# The Performance Characteristics of Handheld, Non-Piezoelectric Point-of-Care Ultrasound (POCUS) in the Emergency Department

**DOI:** 10.3390/diagnostics14010017

**Published:** 2023-12-21

**Authors:** Brandon Michael Wubben, Hae In Yun

**Affiliations:** 1 Department of Emergency Medicine, University of Iowa, 200 Hawkins Drive, Iowa City, IA 52242, USA; 2 Carver College of Medicine, University of Iowa, 375 Newton Road, Iowa City, IA 52242, USA; hiyun@uiowa.edu

**Keywords:** point-of-care ultrasound, emergency medicine, handheld, non-piezoelectric, echocardiography, pleural effusion, hydronephrosis, cholecystitis

## Abstract

The use of handheld point-of-care ultrasound (HH-POCUS) platforms is rapidly increasing, but the diagnostic performance of HH-POCUS in the emergency department (ED) has not been well-studied. For a period of one year, only a HH-POCUS system that uses a non-piezoelectric array (Butterfly iQ+™) was available for clinical POCUS examinations in our ED. We performed a retrospective observational study of patients who underwent cardiac, thoracic, renal, biliary, or lower extremity venous (DVT) examinations from November 2021–November 2022 and calculated performance characteristics of HH-POCUS relative to radiology imaging. A total of 381 HH-POCUS studies were evaluated. Cardiac image quality was significantly lower than lung (*p* = 0.002). Over half of the studies (213/381) had imaging available for comparison, and HH-POCUS identified 86.5% (32/37, (95%CI) 70.4–94.9) of prespecified emergent diagnoses, including acute cholecystitis, severely reduced left ventricular ejection fraction, pericardial effusion or tamponade, moderate or larger pleural effusion, pneumothorax, moderate or larger hydronephrosis, and DVT. For less emergent diagnoses, 84.3% (43/51, (95%CI) 70.9–92.5) were identified. Overall, HH-POCUS using a non-piezoelectric array showed modest real-world performance in the ED for cardiac, thoracic, renal, biliary, and DVT examinations. HH-POCUS may be inadequate to rule out some common ED diagnoses, but had good specificity for certain conditions such as pericardial effusion.

## 1. Introduction

The availability and use of handheld point-of-care ultrasound systems (HH-POCUS) has grown rapidly throughout prehospital emergency care and hospital medicine, and has expanded widely across specialty lines [1,2,3,4,5,6,7]. Emergency medicine clinicians have been quick to adopt point-of-care ultrasound (POCUS) technology, and the benefits of POCUS in the emergency care setting, such as faster intervention for patients with cardiac tamponade or ruptured ectopic pregnancy, have since been demonstrated [8,9]. While many emergency care centers have access to cart-based POCUS systems, over the past few years the use of HH-POCUS has been expanding [1]. Societies such as the American College of Emergency Physicians have published appropriate use criteria for handheld ultrasound in the emergency department (ED), citing earlier studies that suggest HH-POCUS image quality was comparable to conventional machines [10]. Although several HH-POCUS platforms have performed favorably when compared directly to cart-based ultrasound systems for specific use cases [11,12,13,14], and qualitative reports by emergency physicians working in a rural African EDs have suggested that HH-POCUS has a high clinical utility [15], the real-world day-to-day performance of newer non-piezoelectric HH-POCUS systems in the ED has not been well-studied [1].

For a period of one year, instead of our previously-used cart-based POCUS systems, only a non-piezoelectric HH-POCUS system (Butterfly iQ+, Butterfly Network Inc., Burlington, MA, USA) was available for POCUS examinations in the ED. The objective of the current study was to characterize the diagnostic performance characteristics of HH-POCUS in the ED during the study period, with a secondary objective of describing performance for prespecified emergent versus less emergent diagnoses.

## 2. Materials and Methods

This was a retrospective, observational cohort study at a Level 1 trauma and emergency care center with greater than 50,000 annual patient visits. The site was a single academic teaching hospital affiliated with a medical school and a three-year emergency medicine (EM) residency program. A variety of medical, surgical, and obstetric specialties also provide consultative care in the ED. There was no advanced emergency ultrasound fellowship in place at the time of the study, but radiology performed ultrasound (excluding echocardiography) was generally available 24/7. This study was approved by the University Institutional Review Board, including a waiver of informed consent.

Patients who underwent HH-POCUS in the ED between 1 November 2021 and 1 November 2022 using a handheld ultrasound system (Butterfly iQ+, Butterfly Network Inc., Burlington, MA, USA) for clinical patient care were included. First, our emergency ultrasound quality assurance database was queried for patients who underwent cardiac, thoracic, renal, biliary, or lower extremity venous HH-POCUS in the ED during the study period. Second, standardized data abstraction was performed from the electronic medical record and emergency ultrasound quality assurance database using a standardized form hosted on REDCap [16]. A second reviewer reviewed over 10% of the abstracted records for accuracy. Demographic variables and imaging results were recorded including radiology imaging obtained within two days of ED presentation and cardiology imaging obtained within two weeks of ED presentation, to allow for delays in confirmatory cardiology imaging that are common at our institution. The emergency ultrasound quality assurance database was queried for POCUS image quality scores (rated image quality on a Likert scale from 1 (uninterpretable images) to 5 (excellent) with 3 (minimum criteria for diagnosis) as a median by ultrasound fellowship-trained faculty), and POCUS imaging findings were abstracted as described. Cardiac: HH-POCUS left ventricular ejection fraction (LVEF) and the presence of right ventricle dilation were compared to transthoracic echocardiography (TTE) within two weeks of POCUS performance as a gold standard. Ejection fraction categories were: severely reduced (<30%), reduced (30–50%), normal (51–74%), and hyperdynamic (>74%). POCUS was considered to be correct if a range was given for the estimated ejection fraction that overlapped with cardiology-reported LVEF. The reporting worksheet for each HH-POCUS examination requires an LVEF category to be selected rather than an exact percentage specified, as defined above. The detection of overall reduced LVEF was calculated based on the dichotomization of normal (>50%) versus reduced LVEF. For pericardial effusion, POCUS was first compared to TTE (if performed), followed by computed tomography (CT) chest or pericardiocentesis if TTE or CT chest were not performed, respectively. Trivial pericardial effusions were considered to be normal or negative, as “small” was the lowest amount of pericardial fluid able to be indicated on the standardized worksheet (other than the lowest value of “absent”) that clinicians completed for each HH-POCUS examination. Biliary: Scans were first compared to radiology-performed right upper quadrant ultrasound if available, followed by abdominal CT or magnetic resonance imaging (MRI). Lower extremity deep vein thrombosis (DVT): The POCUS standard of care for lower extremity deep veins at our institution is expected to consist of a three-point compression exam including the femoral-saphenous junction, superficial and deep femoral venous junction, and the popliteal vein with approximately 2 cm compressive intervals between the common femoral vein and the popliteal vein; therefore, isolated calf DVT found on full leg duplex ultrasound performed by vascular sonographers were not considered as a comparative finding to HH-POCUS results.

Descriptive analyses were performed regarding the test performance characteristics of HH-POCUS compared to the relevant gold standard as described above. HH-POCUS studies were excluded on a pairwise basis with respect to the absence or presence of specific findings relative to available consultative imaging. For example, for each HH-POCUS echocardiogram, the comparative gold standard imaging may have allowed for the comparison of LVEF, but not RV dilation, depending on the adequacy of the cardiology-performed echocardiogram. Subgroup analyses were performed by the presence or absence of prespecified emergent findings, with emergent findings defined as: severely reduced ejection fraction, pericardial effusion (excluding trivial pericardial effusions), medium or large pleural effusion, pneumothorax, acute cholecystitis, or deep vein thrombosis. Less emergent findings were predefined as: cholelithiasis, reduced left ventricular ejection fraction, small pleural effusion, or mild hydronephrosis. Statistical analysis was performed using SPSS (IBM SPSS Statistics for Macintosh, Version 28. Armonk, NY, USA: IBM Corp).

## 3. Results

**Figure 1:**
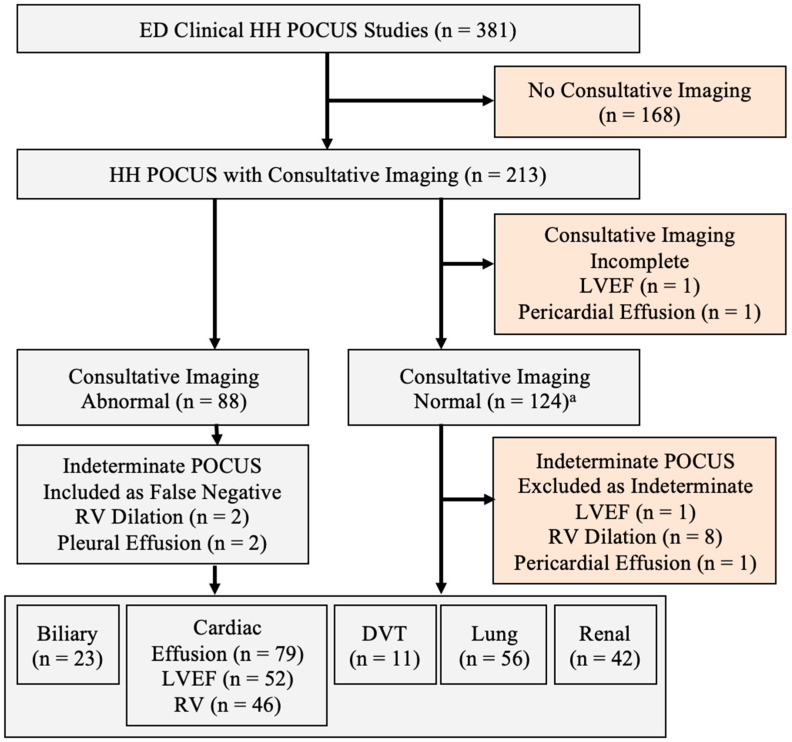
Inclusion diagram for emergency department handheld point-of-care (HH-POCUS) images matched with consultative imaging results. (^a^) Includes two cases where consultative imaging and POCUS were both indeterminate for pleural effusion.

A total of 381 handheld point-of-care ultrasound (HH-POCUS) cardiac, lung, biliary, renal, and lower extremity deep venous thrombosis examinations were performed for clinical patient care in the emergency department between November 2021 and November 2022 (Figure 1).

**Figure 2:**
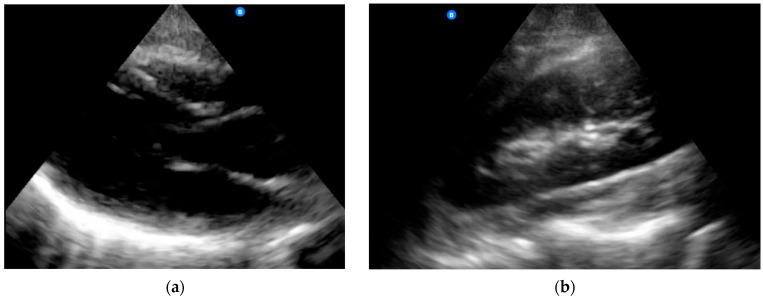
Example images obtained with the Butterfly iQ+ platform: (**a**) normal cardiac parasternal long axis view; (**b**) normal renal long axis view.

The mean patient age was 53.7 years (±21.6, *n* = 380), mean BMI was 29.9 (±7.5, *n* = 263), and 51.2% were female; 77.6% of POCUS studies were signed by an emergency physician who had completed an emergency ultrasound fellowship. As shown in Table 1, the mean POCUS image quality ratings varied significantly by study type (ANOVA *p* = 0.001), with cardiac study quality being significantly lower than lung (*p* = 0.002 with Bonferroni correction). Example images obtained using the Butterfly iQ+ system are shown in Figure 2.

Consultative imaging was available for comparison for 55.9% (213/381) of HH-POCUS studies (Table 2). The prevalence of at least one abnormal finding on consultative imaging was 41.5%. Overall, POCUS was 86.4% ((95%CI) 77.0–92.5) sensitive and 82.3% ((95%CI) 74.1–88.3) specific for the categorization of a study as abnormal when there were abnormal findings on consultative imaging. Test performance characteristics for selected conditions are presented in Table 3.

HH-POCUS was correctly reported as abnormal in 32/37 (86.5%, (95%CI) 70.4–94.9), of studies with consultative imaging identifying one or more diagnoses categorized as emergent including acute cholecystitis, severely reduced left ventricular ejection fraction (LVEF), pericardial effusion, cardiac tamponade, moderate or larger pleural effusion, pneumothorax, moderate or larger hydronephrosis, and deep vein thrombosis. For diagnoses categorized as less emergent, including cholelithiasis, reduced left ventricular ejection fraction, small pleural effusion, or mild hydronephrosis, HH-POCUS was correctly reported as abnormal in 43/51 studies (84.3%, (95%CI) 70.9–92.5).

Including all emergent and less emergent findings, there was complete agreement for all findings between the POCUS result and consultative imaging in 69.3% (147/193, (95%CI) 62.6–75.4). Examples of reasons why complete agreement was not reached included the recognition but incorrect categorization of severity of decreased LVEF, or the correct categorization of LVEF but missed pericardial effusion or missed RV dilation.

## 4. Discussion

Overall, we found that HH-POCUS using a non-piezoelectric system (Butterfly iQ+) had modest real-world performance in the emergency department, with a pooled sensitivity of 86.4% and pooled specificity of 82.3% for detecting abnormalities on cardiac, thoracic, renal, biliary, and lower extremity venous scans. However, HH-POCUS had a high specificity for several emergent medical conditions frequently requiring prompt intervention, such as pericardial effusion, right ventricle dilation, hydronephrosis, and DVT. Our findings suggest that HH-POCUS is a valuable bedside tool for guiding emergency diagnosis and treatment.

Although prior studies have examined the performance of other HH-POCUS platforms such as Vscan (a piezoelectric system) for specific use cases, to our knowledge, the current study is the first to report the longitudinal real-world diagnostic performance of a non-piezoelectric HH-POCUS system in the ED.

### 4.1. Application-Specific Performance

#### 4.1.1. Cardiac

Prior comparative studies have generally found good agreement between other HH-POCUS platforms such as Vscan (GE Healthcare, Chicago, IL, USA) and cart-based machines used by the same type of clinician (internist, cardiologist, etc.) in assessing LVEF and pericardial effusions [13,17]. In our study, when comparing HH-POCUS in the emergency department to cardiology-performed echocardiography, pericardial effusion was identified with a high specificity (95.3%) but lower sensitivity (64.7%), similar to the 99% specificity and 54% sensitivity for moderate or large pericardial effusion demonstrated by internal medicine residents using an OptiGo handheld US (Philips Medical Systems, Andover, MA, USA) compared to cardiology-performed echocardiography [18]. While there was only one patient in our study with cardiac tamponade, HH-POCUS allowed for confirmation of the diagnosis at the bedside followed by emergent pericardiocentesis without waiting for additional consultative imaging to make the diagnosis.

HH-POCUS in our study detected reduced LVEF (<50%) with a sensitivity of 85.7% and a lower specificity of 71.0%, somewhat lower than a 2021 meta-analysis, which demonstrated that both experienced and novice operators could detect reduced LVEF (defined as <45% in this study) with a pooled sensitivity of 88% and 83%, with specificity of 96% and 89%, respectively. However, this meta-analysis included no studies using the Butterfly iQ [2]. The poor sensitivity for detecting RV dilation in our study (33.3%) but high specificity (94.6%) is similar to a study comparing HH-POCUS (VScan, GE Healthcare) to a cart-based system, performed by intensivists (59% sensitivity, 98% specificity) for RV dilation detection [13]. The prior meta-analysis highlighted operator experience as a key factor in reduced LVEF detection [2]. The explanation for the lower performance of HH-POCUS for detecting reduced LVEF and RV dilation in our study despite a high level of operator experience is uncertain, but one possibility is the increased difficulty of determining normal versus slightly decreased LVEF based on the use of different cutoff values for normal versus abnormal LVEF (<45% in some studies versus <50% in our study). Our study was also not designed to determine the degree of inaccuracy from a percentage standpoint for LVEF; determinations of LVEF may have differed by 5% or 20%, but accuracy was based on the correct categorical selection of the LVEF category as normal, reduced, or severely reduced. This study design was based on the limitations of the HH-POCUS worksheet completed with each examination. Other factors such as lower cardiac image quality using the Butterfly iQ+ platform and a delay between HH-POCUS and cardiology echocardiogram (with a presumed change in patient condition in the interim) may also have contributed.

#### 4.1.2. Biliary and DVT

The high sensitivity of HH-POCUS for cholelithiasis, acute cholecystitis, and proximal DVT in our study was similar to findings from prior studies using Vscan for detecting cholelithiasis and proximal DVT [19,20]. The small number of positive scans in our study limits the generalizability of conclusions about how HH-POCUS might perform in a higher-volume environment and is an area for further study. However, our center’s performance with these applications appears promising in multiple respects, as in our experience ambulatory patients are frequently referred to emergency care to rule out acute cholecystitis or deep vein thrombosis. HH-POCUS might be able to quickly rule out these conditions. In addition, in unstable patients in the emergency and critical care settings, the ability to quickly evaluate for evidence of DVT with reasonable sensitivity in the setting of undifferentiated hypoxia or cardiac arrest is valuable.

#### 4.1.3. Renal

The lower sensitivity of 73.7% that we observed for detecting hydronephrosis using HH-POCUS is similar to the results from prior studies using Vscan, which report sensitivities ranging from 67% for internal medicine residents to 91% for skilled sonographers in a non-emergent setting [21,22]. However, the specificity for detecting moderate or greater hydronephrosis was excellent, suggesting difficulty in detecting subtle findings, but high diagnostic utility when findings are clearly abnormal. HH-POCUS is likely to be helpful when screening for causes of undifferentiated abdominal or flank pain, as a prior meta-analysis demonstrated that the presence of moderate or greater hydronephrosis had both diagnostic and prognostic value in the evaluation for nephrolithiasis in the emergent setting [23].

#### 4.1.4. Lung

HH-POCUS using Butterfly iQ has previously been studied for specific applications in lung ultrasound, such as examining lung ultrasound scores in COVID-19 patients, where no significant disparities between the scores obtained with Butterfly iQ and a cart-based system were found [12]. We observed a low sensitivity of 62.5% for pleural effusion detection, which contrasts with previous studies reporting up to 100% sensitivity and good overall agreement with cart-based machines for HH-POCUS using Vscan [11,24,25,26]. It is unclear whether this difference was due to disparities in the lung ultrasound zones imaged for each patient, versus the limitations of the probe itself. We included our reported performance for alveolar interstitial syndrome (AIS), which was lower than the previously reported sensitivity of 85.7% and specificity of 97.7% using a GS 50 (Siemens, Germany) compared to CXR [27]. However, while AIS has characteristic findings for ultrasound that generally correlate well with CXR findings, there were a few cases in the prior study where an AIS pattern was present on POCUS but not on CXR, raising mild concern about the suitability of CXR as a gold standard (i.e., we theorize that POCUS may actually be a better test for AIS in some cases than single view CXR). In addition, the HH-POCUS worksheet only allows for the reporting of focal versus diffuse AIS, without options to specify the severity of findings in any particular lung zone. The authors postulate that the performance of HH-POCUS would improve if focusing on the discriminatory performance of severe AIS or confluent B-lines, rather than a dichotomy at the minimum threshold to characterize AIS as present, as the spectrum of lung disease manifested by the varied severity of lung POCUS findings ranges widely based on the degree and severity of involvement [12].

Only one patient in our study had a pneumothorax, which was missed by HH-POCUS. This was a small apical pneumothorax that did not require tube thoracostomy; based on the location, we suspect this miss was secondary to the number of lung fields visualized, rather than a limitation of the device itself.

### 4.2. Platform-Specific Performance

Factors such as varying operator skill level, operator specialty, and varying gold standard imaging modalities ranging from radiology-performed ultrasound to CT make direct comparisons to prior studies more challenging. Additionally, despite the common description of handheld ultrasound as a portable and user-friendly technology, device capabilities vary by manufacturer and the type of technology used for image generation [28]. Our study utilized Butterfly iQ+, which uses a capacitive micromachined membrane to generate images instead of the piezoelectric crystals that have been traditionally used. Butterfly iQ+ and other similar non-piezoelectric probes include only a single transducer that emulates the piezoelectric crystal arrays of other probe types based on selected application and depth. Other HH-POCUS platforms may offer multiple probes with distinct advantages and limitations [1,28]. Cardiac image quality in our study was relatively low; in our practical experience, the large transducer footprint of the Butterfly iQ+ is difficult to place between small rib spaces, and often gathers artifacts from surrounding lung tissue. A prior qualitative study in an austere ED also noted this difficulty [15]. In a prior review of several different HH-POCUS systems by POCUS experts, Butterfly iQ+ was consistently rated below three other platforms (including Vscan) with respect to image quality, including detail resolution, penetration, clutter, and overall satisfaction [29]. A further study is needed to determine if POCUS using a piezoelectric HH-POCUS system would offer similar longitudinal performance to what was observed in the current study, as other types of HH-POCUS were not available for use in the ED during our study period. While pediatric patients were included in our study, the majority of HH-POCUS examinations were performed on adults. Based on our experience, we suspect that the large footprint of the non-piezoelectric transducer may make image acquisition more difficult with younger children who have small windows. Further studies are needed to determine if HH-POCUS is suitable in this patient population. Future studies should continue to explore the use of this technology in less resource-rich settings, such as a promising prior study on the use of the Butterfly iQ platform by paramedics in out-of-hospital cardiac arrest, and a proof-of-concept study for the use of HH-POCUS in hyperbaric chambers [30,31].

### 4.3. Limitations

As a retrospective study, we identified patients who had undergone HH-POCUS as documented in the medical record, but it is possible that some patients received undocumented HH-POCUS as part of their care. If poor-quality images that were discarded were instead submitted, this could decrease the performance characteristics of HH-POCUS. However, it is the standard of care at our institution to save all images used for clinical care in the medical record. Second, alternative imaging modalities may have been obtained prior to the patient’s arrival to the ED. In such cases, the EP conducting the scan may have been aware of the findings derived from the previous imaging, potentially introducing positive bias into the interpretation of the results. However, we believe that in the majority of cases, POCUS was performed before obtaining other imaging. Our study design does not allow for direct comparisons of how cart-based POCUS systems or other types of HH-POCUS systems would have performed in the same longitudinal circumstances, and our study does not conclude on whether non-piezoelectric HH-POCUS is better or worse than other POCUS types. However, our study does provide real-world data about the longitudinal performance that might be expected if used routinely in the emergency care setting.

## 5. Conclusions

Over the one-year period where only a non-piezoelectric HH-POCUS system was available for clinical patient POCUS examinations in our ED, HH-POCUS showed modest real-world diagnostic performance for cardiac, thoracic, renal, biliary, and lower extremity venous scans. HH-POCUS was highly specific for pericardial effusion, right ventricle dilation, moderate or greater hydronephrosis, and DVT, allowing clinicians to rule in these diagnoses at the bedside in the emergent and critical care setting. However, the limited sensitivity of HH-POCUS for pleural effusion, right ventricle dilation, and mild hydronephrosis should encourage increased caution if being used as a stand-alone test, as HH-POCUS may be insufficient to rule out these diagnoses. Overall, HH-POCUS is a valuable tool for emergency department patient care.

## Figures and Tables

**Table 1:** The mean image quality assurance score by study type for handheld point-of-care ultrasound using Butterfly iQ in the emergency department. DVT = deep vein thrombosis.

Study Type	Mean Quality Assurance Score
Biliary	3.44 ± 0.89
Cardiac ^a^	3.41 ± 0.81
DVT	3.77 ± 0.89
Lung ^a^	3.87 ± 0.78
Renal	3.53 ± 0.72

^a^ Significant differences (*p* = 0.002) when adjusted for multiple comparisons.

**Table 2:** Consultative imaging type used for comparison to HH-POCUS studies. (*n*) = number of POCUS studies applied to comparison; RUQ = right upper quadrant; US = ultrasound; MRI = magnetic resonance imaging; TTE = transthoracic echocardiography; CT = computed tomography; LVEF = left ventricular ejection fraction; DVT = deep vein thrombosis; CXR = chest X-ray.

Scheme	Comparison
Biliary (*n* = 23)	RUQ US (*n* = 11)
	CT Abdomen (*n* = 10)
	Abdominal MRI (*n* = 2)
Pericardial Effusion (*n* = 79)	TTE (*n* = 53)
	CT Chest (*n* = 25)
	Pericardiocentesis (*n* = 1)
Right Ventricle (*n* = 46)	TTE (*n* = 46)
LVEF (*n* = 52)	TTE (*n* = 52)
DVT (*n* = 11)	Lower Extremity Duplex Ultrasound (*n* = 11)
Lung (*n* = 56)	CT Chest (*n* = 22)
	CXR (*n* = 34)
Renal (*n* = 42)	CT Abdomen (*n* = 42)

**Table 3:** The performance characteristics of handheld point-of-care ultrasound using Butterfly iQ in the emergency department compared to consultative imaging. LVEF = left ventricular ejection fraction; RV = right ventricle; DVT = deep vein thrombosis.

Finding	Identified/Positives	Sensitivity (95%CI)	Specificity (95%CI)
Acute cholecystitis	1/1	100 (5.0–100)	95.5 (75.1–99.8)
Uncomplicated cholelithiasis	3/3	100 (31.0–100)	88.9 (63.9–98.1)
Reduced LVEF	18/21	85.7 (62.6–96.2)	71.0 (51.8–85.1)
LVEF category	16/22	72.7 (49.6–88.4)	56.7 (37.7–74.0)
Pericardial effusion	11/17	64.7 (38.6–84.7)	96.8 (87.8–99.4)
Dilated RV	3/9	33.3 (9.0–69.1)	94.6 (80.5–99.1)
Cardiac tamponade	1/1	-	-
DVT present	3/3	100 (31.0–100)	100 (59.8–100)
Alveolar interstitial syndrome	8/11	72.7 (39.3–92.7)	62.2 (46.5–75.8)
Pleural effusion	15/24	62.5 (40.8–80.4)	90.6 (73.8–97.5)
Pneumothorax	0/1	-	-
≥Moderate hydronephrosis	4/5	80.0 (29.9–98.9)	97.3 (84.2–99.9)
Any hydronephrosis	14/19	73.7 (48.6–89.9)	87.0 (65.3–96.6)

## Data Availability

The data presented in this study are available on a limited basis on request from the corresponding author. The data are not publicly available due to patient privacy restrictions requiring the minimum sharing necessary.
